# Supplementation of melatonin to cooling and freezing extenders improves canine spermatozoa quality measures

**DOI:** 10.1186/s12917-022-03186-8

**Published:** 2022-03-05

**Authors:** Mohammad Reza Divar, Mehdi Azari, Asghar Mogheiseh, Sadegh Ghahramani

**Affiliations:** 1 Department of Clinical Sciences, School of Veterinary Medicine, Shiraz University, P.O.BOX: 7144169155, Fars Shiraz, Iran; 2 DVM Candidate, School of Veterinary Medicine, Shiraz University, Shiraz, Fars Iran

**Keywords:** Sperm, Dog, Cooling, Freezing, Melatonin

## Abstract

**Background:**

Sperm freezing and cold storage are the two most common assisted reproductive technologies in the canine breeding industry. The freeze-thawing process causes significant detrimental changes in both sperm cell structure and function. Previous research has confirmed that excessive accumulation of un-scavenged free radicals (oxidative stress) plays an important role in the cryopreservation-induced damage to sperm cells. Also, the gradual accumulation of the free radicals during cold storage leads to a decline in the sperm quality markers. Melatonin is an endogenous neurohormone synthesized from tryptophan amino acid by pineal glands. Besides its several well-known physiologic roles, melatonin has a significant antioxidant potential through direct free radical scavenging properties. Therefore, the current study was designed to evaluate the potential in vitro protective properties of melatonin (0.5, 1, and 2 mM) on canine sperm cells after freezing or during long-term cold storage (9 days, 5 °C) on most important sperm in vitro fertility markers.

**Results:**

Melatonin at 0.5, 1- or 2-mM concentrations could preserve significantly higher sperm total motility after 4 days of cold storage. However, only the 1- and 2 mM melatonin concentrations could result in better TM and PM values after 7 days of cold storage. Furthermore, melatonin supplementation could preserve higher sperm viability and acrosome integrity after 7 days of storage. Also, it could have significant protective effects on the cooled sperm DNA integrity. In the freezing section of the current research, melatonin at either 1- or 2-mM concentrations could not improve the sperm post-thaw TM and PM, whereas they improved sperm DNA integrity. Also, the post-thaw plasma membrane functional integrity and sperm velocity parameters were not affected by the treatment. Although DMSO (Dimethyl Sulfoxide) as the melatonin solvent could reduce the level of sperm lipid peroxidation and even improve the post-thaw sperm DNA integrity compared to the negative control, it reduced the post-thaw sperm progressive motility. However, the negative effects were reversed by concurrent melatonin supplementation at 1- and 2-mM concentrations.

**Conclusion:**

The addition of 1- or 2-mM melatonin to the canine sperm freezing and cooling media could improve sperm motility, viability, acrosome, and DNA integrity.

## Background

Successful cryopreservation of the spermatozoa with acceptable post-thaw quality measures paves the way for effective conservation and distribution of the outstanding genetic potentials in the canine breeding industry [1]. Furthermore, cold storage (5 °C) can successfully extend the longevity of canine spermatozoa for several days (up to 14 days) which makes it possible to transfer fertile semen between distant geographic locations [2]. It also enables the transfer of the semen collected at the male stud location to a laboratory equipped with freezing utilities without deterioration of sperm freezability [3]. However, both the freeze-thawing and the long-term cold storage cause significant functional and structural disturbances in the sperm cells [4]. The detailed pathophysiology of the sperm cold shock and cryo-injury has been subjected to extensive research during recent years [5].

Along with other mechanisms, oxidative stress, defined as excessive accumulation of the un-scavenged free oxygen and nitrogen radicals, has been proved to play a central role in sperm damage during both cold storage and after cryopreservation [6]. Free radicals are highly reactive and attack many cellular targets leading to irreversible impairments. In confirmation, many studies have shown the positive in vitro effects of the several enzymatic or non-enzymatic antioxidants in lessening the negative effects of oxidative stress during cryopreservation of sperm cells in multiple animal species [7].

Melatonin is an endogenous neurohormone synthesized from tryptophan amino acid by pineal glands. Besides its several well-known physiologic roles, melatonin has a significant antioxidant potential through direct free radical scavenging properties. It may also exert indirect stimulatory effects on several antioxidant enzymes such as glutathione peroxidase/reductase, catalase, and superoxide dismutase [8]. The protective effects of melatonin supplementation of the cryopreservation media on the porcine, bovine, ovine, equine and, human frozen-thawed spermatozoa have been previously reported in several independent studies [9]. Canine frozen-thawed ejaculated [10] or epididymal [11] spermatozoa have also benefited from in vitro supplementation of the melatonin in the freezing extender. However, multiple studies show that the extent of the protective effects is species-specific and highly dependent on the melatonin concentration.

Based on the available literature, the in vitro potential protective effects of melatonin and its optimum concentration on canine frozen-thawed ejaculated spermatozoa are not explored well. Also, the protective effects of melatonin on the canine spermatozoa under cold storage are not investigated yet. Therefore, the current research was designed to evaluate the effects of melatonin supplementation of the freezing and cooling media at different levels (0.5, 1, and 2 mM) on canine spermatozoa in vitro fertility markers during long-term cold storage (9 days, 5 °C) and after cryopreservation.

## Results

### Effects of melatonin supplementation on canine spermatozoa under cold storage (9 days, 5 °C)

#### Sperm motility parameters

**Fig. 1:**
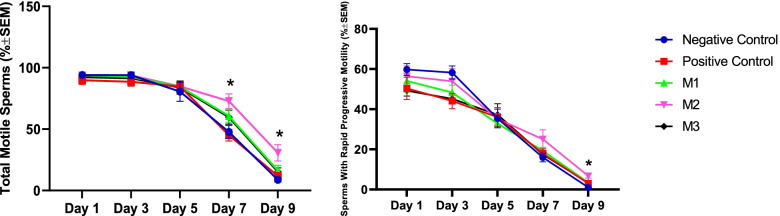
Changes in the total (TM) and progressive (PM) canine sperm motility during cold storage (5 °C) in extenders containing different levels of melatonin versus the controls. A two-way repeated-measures Analysis of Variance (ANOVA) statistical test was used to make a comparison between different levels of melatonin versus the control at different times. Pairwise comparisons with Least Significant Difference (LSD) confidence interval adjustments were carried out to examine the simple effects for the time and study groups. Negative Control (without Melatonin or DMSO), Positive Control (containing DMSO), M1 (containing 0.5 mM Melatonin), M2 (containing 1 mM Melatonin), and M3 (containing 2 mM Melatonin). All numbers are presented as mean’s ± SEM. The asterisks show a statistically significant difference (*p* ≤ 0.05) between the M2 group compared with the other groups at the specified time points

The percent of the total (TM) and rapid-progressive (PM) motile spermatozoa remained unchanged during the first 48 h (Days 1 and 3) of the cold storage (5 °C) in all study groups. However, the TM and PM values showed a significant decline over the last 96 h (Days 7 and 9) in all groups. On the Day 5 of the cold storage, DMSO (Dimethyl Sulfoxide) alone or with melatonin at all studied concentrations could significantly improve the sperm TM compared to the negative control. However, the Day 5 PM values remained unaffected. At Days 7 and 9, melatonin at 1 mM concentration could preserve higher TM values compared with the other groups. Also, melatonin at the 1 mM level could improve the sperm PM at Day 9 of the study (Fig. 1).

From Day 7, sperm velocity parameters (Velocity Curvilinear; VCL, Velocity Average Path; VAP, and Velocity Straight Line; VSL) showed a significant decrease in all study groups. The melatonin supplementation at 1 mM could improve the VCL and VSL values only at Day 9 of the cold storage compared with other groups (Table 1). Similarly, the velocity ratios (Straightness; STR, Linearity; LIN, and Wobble; WOB) decreased significantly from Day 7 onwards in all groups. The melatonin at 1 mM could improve the LIN value only at Day 9 of the cold storage compared with other groups (Table 2). Other velocity parameters (Amplitude of Lateral Head Displacement; ALH, Beat Cross Frequency; BCF, and Mean Angular Displacement; MAD) also showed a significant decline from Day 7 of the cold storage. Overall, at Days 7 and 9, Melatonin at 1 mM concentration could preserve higher sperm motion velocity values compared with the others (Table 3).

**Table 1:** Changes in the canine sperm velocity parameters (μm/s) during cold storage (5 °C) in the extenders containing different levels of melatonin versus the controls

Sampling Days	Group				
	Negative Control	Positive Control	M1	M2	M3
**VCL1**	70.36 ± 4.40 ^1^	66.42 ± 6.11 ^1^	67.15 ± 4.21 ^1^	65.84 ± 5.36 ^1^	68.72 ± 8.44 ^12^
**VCL3**	65.55 ± 4.75 ^1^	62.50 ± 6.67 ^1^	63.88 ± 4.47 ^1^	63.53 ± 5.16 ^1^	67.04 ± 8.42 ^1^
**VCL5**	53.22 ± 6.49 ^2^	52.22 ± 4.44 ^1^	53.33 ± 1.38 ^1^	52.59 ± 2.26 ^1^	53.10 ± 5.01 ^2^
**VCL7**	23.08 ± 2.14 ^2^	31.05 ± 5.51 ^2^	28.84 ± 2.70 ^2^	34.18 ± 3.65 ^2^	27.19 ± 3.29 ^3^
**VCL9**	3.19 ± .62 ^3a^	4.66 ± .69 ^2a^	6.55 ± 1.73 ^3abc^	9.57 ± 1.99 ^3bc^	6.06 ± 1.22 ^4ac^
**VAP1**	39.59 ± 3.05 ^1^	35.56 ± 4.06 ^1^	38.51 ± 4.17 ^1^	36.45 ± 3.39 ^12^	36.40 ± 4.39 ^12^
**VAP3**	35.91 ± 1.60 ^1^	31.32 ± 3.70 ^1^	33.48 ± 2.22 ^1^	34.56 ± 2.60 ^2^	34.24 ± 3.95 ^2^
**VAP5**	25.64 ± 2.99 ^2^	25.83 ± 2.09 ^1^	25.63 ± .62 ^1^	26.28 ± 1.31 ^1^	26.14 ± 2.23 ^1^
**VAP7**	11.04 ± .95 ^23^	14.36 ± 2.64 ^2^	13.41 ± 1.19 ^2^	15.94 ± 1.96 ^3^	13.00 ± 1.48 ^3^
**VAP9**	1.34 ± .33 ^3^	2.09 ± .26 ^3^	2.92 ± .88 ^3^	4.77 ± 1.06 ^4^	2.74 ± .63 ^4^
**VSL1**	29.37 ± 2.94 ^1^	24.29 ± 3.37 ^123^	28.70 ± 3.61 ^1^	26.64 ± 2.94 ^12^	25.91 ± 3.23 ^1^
**VSL3**	26.39 ± .08 ^1b^	20.86 ± 2.57 ^12a^	24.23 ± 1.90 ^12ab^	25.14 ± 1.94 ^1ab^	24.08 ± 2.61 ^1ab^
**VSL5**	17.27 ± 1.75 ^2^	17.32 ± 1.44 ^12^	16.63 ± .81 ^2^	17.78 ± 1.10 ^2^	18.09 ± 1.54 ^1^
**VSL7**	6.99 ± .50 ^23^	8.96 ± 1.77 ^3^	8.37 ± .73 ^3^	10.10 ± 1.41 ^3^	8.11 ± .88 ^3^
**VSL9**	.71 ± .18 ^3a^	1.25 ± .18 ^4a^	1.65 ± .53 ^4acb^	3.02 ± .71 ^4c^	1.51 ± .41 ^4ab^

The different numbers and letters in superscripts indicate a statistically significant difference (P ≤ 0.05) within and between columns, respectively. A two-way repeated-measures ANOVA statistical test was used to make a comparison between treatment groups at different times. Pairwise comparisons with LSD confidence interval adjustments were carried out to examine the simple effects for the time and study groups. Negative Control (without Melatonin or DMSO), Positive Control (containing DMSO), M1 (containing 0.5 mM Melatonin), M2 (containing 1 mM Melatonin), and M3 (containing 2 mM Melatonin). All numbers are presented as Means±SEM. *VCL* Velocity Curvilinear, *VAP* Velocity Average Path, and *VSL* Velocity Straight Path

**Table 2:** Changes in the canine sperm velocity ratios (%) during cold storage (5 °C) in the extenders containing different levels of melatonin versus the controls

Sampling Days	Group				
	Negative Control	Positive Control	M1	M2	M3
**STR1**	74.03 ± 3.16 ^1b^	67.57 ± 2.15 ^12a^	74.08 ± 1.95 ^1ab^	72.74 ± 2.16 ^1ab^	71.12 ± 1.55 ^1ab^
**STR3**	73.90 ± 3.08 ^12bc^	66.51 ± 1.40 ^12a^	72.17 ± 1.86 ^12ac^	72.78 ± 2.20 ^1c^	70.58 ± 1.18 ^1ac^
**STR5**	67.90 ± 1.21 ^23^	67.15 ± 1.96 ^2^	64.81 ± 2.14 ^2^	67.52 ± 1.04 ^23^	69.24 ± 1.31 ^1^
**STR7**	63.63 ± 1.19 ^3^	61.88 ± 1.54 ^13^	62.50 ± .83 ^23^	62.84 ± 1.36 ^3^	62.60 ± 1.05 ^23^
**STR9**	52.44 ± .86 ^4^	59.60 ± 2.18 ^3^	55.13 ± 2.19 ^3^	62.82 ± 1.58 ^3^	54.01 ± 5.12 ^3^
**LIN1**	41.89 ± 3.53 ^12^	36.27 ± 3.26 ^12^	42.59 ± 4.18 ^1^	40.33 ± 2.62 ^12^	38.04 ± 2.61 ^1^
**LIN3**	40.88 ± 2.85 ^2bc^	33.24 ± 1.42 ^12a^	37.97 ± 1.86 ^1ac^	39.77 ± 2.12 ^2c^	36.41 ± 1.66 ^1ac^
**LIN5**	32.82 ± .76 ^13^	33.35 ± 1.48 ^2^	31.23 ± 1.53 ^123^	33.74 ± 1.05 ^134^	34.29 ± 1.35 ^2^
**LIN7**	30.52 ± .81 ^13^	28.53 ± 1.39 ^3^	29.12 ± .49 ^3^	29.13 ± 1.07 ^34^	30.00 ± .52 ^13^
**LIN9**	21.56 ± 1.80 ^4ab^	26.87 ± 1.45 ^34abc^	24.12 ± 2.58 ^34ab^	31.12 ± 1.80 ^4c^	24.06 ± 2.62 ^3b^
**WOB1**	56.30 ± 2.43 ^12^	53.29 ± 3.01 ^12^	57.15 ± 4.36 ^1^	55.24 ± 1.93 ^1^	53.28 ± 2.49 ^1^
**WOB3**	55.12 ± 1.56 ^1b^	49.91 ± 1.41 ^124a^	52.49 ± 1.22 ^12ab^	54.51 ± 1.24 ^12ab^	51.50 ± 1.62 ^1ab^
**WOB5**	48.31 ± .33 ^234^	49.59 ± .89 ^2^	48.08 ± .77 ^2^	49.92 ± .85 ^13^	49.45 ± 1.05 ^1^
**WOB7**	47.94 ± .38 ^34^	46.02 ± 1.29 ^134^	46.59 ± .42 ^23^	46.30 ± .80 ^14^	47.94 ± .34 ^12^
**WOB9**	41.02 ± 2.82 ^4^	45.06 ± 1.41 ^4^	43.55 ± 3.21 ^3^	49.46 ± 1.60 ^234^	44.50 ± 1.97 ^2^

The different numbers and letters in superscripts indicate a statistically significant difference (P ≤ 0.05) within and between columns, respectively. A two-way repeated-measures ANOVA statistical test was used to make a comparison between treatment groups at different times. Pairwise comparisons with LSD confidence interval adjustments were carried out to examine the simple effects for the time and study groups. Negative Control (without Melatonin or DMSO), Positive Control (containing DMSO), M1 (containing 0.5 mM Melatonin), M2 (containing 1 mM Melatonin), and M3 (containing 2 mM Melatonin). All numbers are presented as Means±SEM. *STR* Straightness, *LIN* Linearity, and *WOB* Wobble

**Table 3:** Changes in the canine sperm velocity parameters during cold storage (5 °C) in the extenders containing different levels of melatonin versus the controls

Sampling Days	Group				
	Negative Control	Positive Control	M1	M2	M3
**ALH1**	3.41 ± .20 ^1^	3.28 ± .21 ^1^	3.33 ± .16 ^1^	3.23 ± .25 ^1^	3.34 ± .38 ^1^
**ALH3**	3.34 ± .23 ^1^	3.26 ± .23 ^1^	3.27 ± .17 ^1^	3.15 ± .26 ^1^	3.36 ± .38 ^1^
**ALH5**	2.74 ± .31 ^2^	2.82 ± .22 ^1^	2.91 ± .12 ^1^	2.84 ± .11 ^12^	2.86 ± .25 ^12^
**ALH7**	1.40 ± .14 ^2a^	1.89 ± .32 ^2ab^	1.78 ± .18 ^2ab^	2.12 ± .18 ^2b^	1.72 ± .19 ^3ab^
**ALH9**	.25 ± .05 ^3a^	.35 ± .05 ^3a^	.47 ± .10 ^3a^	.76 ± .12 ^3b^	.44 ± .07 ^4a^
**BCF1**	5.04 ± .17 ^1^	4.33 ± .21 ^1^	4.43 ± .20 ^1^	4.60 ± .10 ^1^	4.53 ± .10 ^1^
**BCF3**	4.99 ± .20 ^1^	4.43 ± .17 ^1^	4.45 ± .20 ^1^	4.78 ± .22 ^1^	4.54 ± .11 ^1^
**BCF5**	3.91 ± .26 ^23^	4.36 ± .26 ^12^	4.12 ± .21 ^12^	4.33 ± .05 ^1^	4.22 ± .42 ^1^
**BCF7**	2.22 ± .24 ^34a^	3.10 ± .54 ^2ab^	3.08 ± .36 ^23ab^	3.93 ± .33 ^1c^	2.94 ± .31 ^2ab^
**BCF9**	.33 ± .05 ^4a^	.44 ± .08 ^3a^	.57 ± .15 ^4ab^	1.00 ± .25 ^2b^	.56 ± .18 ^3ab^
**MAD1**	66.68 ± 4.62 ^1^	66.18 ± 4.68 ^1^	66.87 ± 2.66 ^1^	64.70 ± 3.77 ^1^	68.97 ± 5.47 ^1^
**MAD3**	63.39 ± 5.66 ^1^	66.20 ± 4.68 ^1^	66.06 ± 2.74 ^1^	64.70 ± 3.77 ^1^	69.93 ± 5.33 ^1^
**MAD5**	60.47 ± 6.78 ^1^	60.87 ± 4.32 ^1^	57.18 ± 3.86 ^23^	60.40 ± 2.78 ^23^	57.06 ± 6.01 ^2^
**MAD7**	27.80 ± 3.39 ^2a^	39.20 ± 7.37 ^2ab^	36.61 ± 2.86 ^3ab^	44.91 ± 4.53 ^3b^	34.25 ± 4.81 ^3ab^
**MAD9**	3.99 ± .63 ^3a^	6.28 ± 0.76 ^3ab^	8.67 ± 2.26 ^4ab^	11.16 ± 1.91 ^4b^	8.36 ± 1.81 ^4ab^

The different numbers and letters in superscripts indicate a statistically significant difference (P ≤ 0.05) within and between columns, respectively. A two-way repeated-measures ANOVA statistical test was used to make a comparison between treatment groups at different times. Pairwise comparisons with LSD confidence interval adjustments were carried out to examine the simple effects for the time and study groups. Negative Control (without Melatonin or DMSO), Positive Control (containing DMSO), M1 (containing 0.5 mM Melatonin), M2 (containing 1 mM Melatonin), and M3 (containing 2 mM Melatonin). All numbers are presented as Means±SEM. *ALH* Amplitude of Lateral Head Displacement (μm), *BCF* Beat Cross Frequency (Hz), and *MAD* Mean Angular Displacement (°)

#### Sperm viability and morphology

**Fig. 2:**
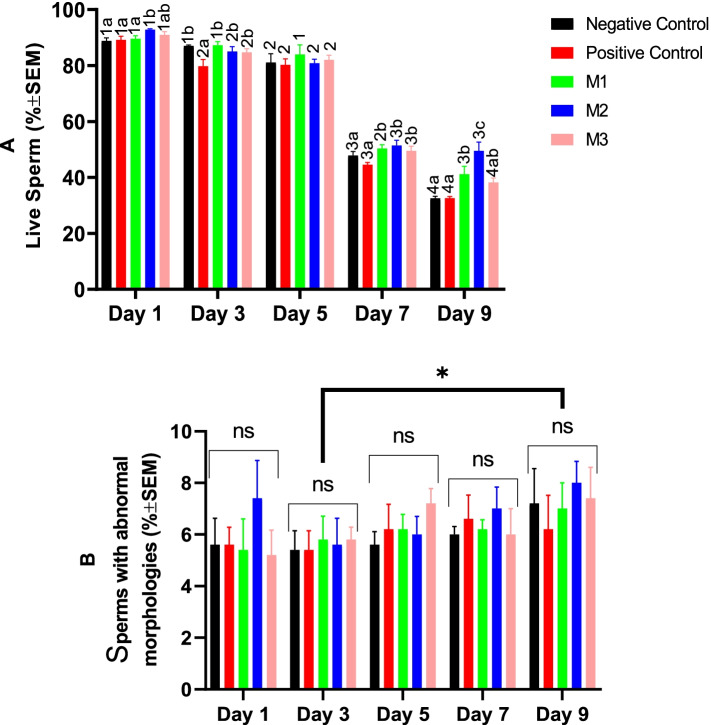
Changes in the canine sperm viability and abnormal morphology during cold storage (5 °C) in the extenders containing different levels of melatonin versus the controls. A two-way repeated-measures ANOVA statistical test was used to make a comparison between treatment groups at different times. Pairwise comparisons with LSD confidence interval adjustments were carried out to examine the simple effects for the time and study groups. The different numbers and letters in the bar superscripts indicate a statistically significant difference (*P* ≤ 0.05) between the time points in each study group and between the study groups at each time point, respectively. The asterisk shows a statistically significant difference (P ≤ 0.05) between time points. Control (without Melatonin or DMSO), Positive Control (containing DMSO), M1 (containing 0.5 mM Melatonin), M2 (containing 1 mM Melatonin), and M3 (containing 2 mM Melatonin), ns, non-significant. All numbers are presented as means±SEM

The percent of the viable spermatozoa showed only a marginal but statistically significant decrease during the first 96 h of the cold storage almost in all study groups. However, from Day 7 a considerable significant reduction was observed in all groups. On day 7, melatonin supplementation at all studied levels could significantly improve sperm viability, while the DMSO alone could not. On Day 9 of the study melatonin at the second level (1 mM) could preserve the sperm viability higher than other groups, significantly (Fig. 2A). The percent of the sperms with abnormal morphologies was not affected by the study groups throughout the cold storage. However, it increased significantly at Day 9 compared with Day 2 (Fig. 2B).

### Sperm membrane functional integrity

**Fig. 3:**
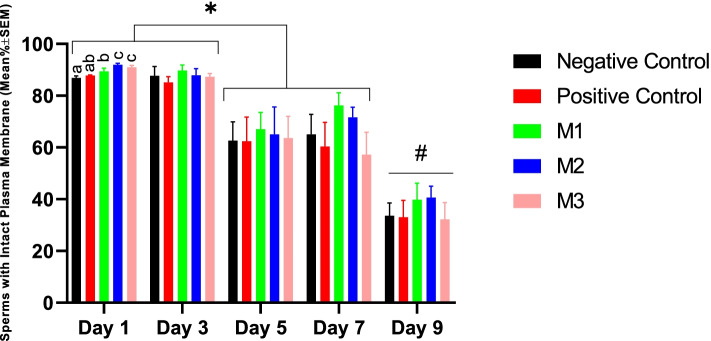
Changes in canine sperm plasma membrane functional integrity during cold storage (5 °C) in the extenders containing different levels of melatonin versus the control. A two-way repeated-measures ANOVA statistical test was used to make a comparison between treatment groups at different times. Pairwise comparisons with LSD confidence interval adjustments were carried out to examine the simple effects for the time and study groups. The different letters in bar superscripts indicate a statistically significant difference (*P* ≤ 0.05) between study groups at the specified time point. The number sign shows a statistically significant difference (P ≤ 0.05) between Day 9 and other time points. The asterisk shows a statistically significant difference (P ≤ 0.05). Negative Control (without Melatonin or DMSO), Positive Control (containing DMSO), M1 (containing 0.5 mM Melatonin), M2 (containing 1 mM Melatonin), and M3 (containing 2 mM Melatonin), ns, non-significant. All numbers are presented as Means±SEM

The first and the second statistically significant declines in the biochemical integrity of the sperm plasma membrane were noticed respectively at Day 5 and Day 9 of the cold storage in all study groups. On Day 1, the M2 and M3 groups showed significantly higher values for sperm plasma membrane functional integrity (*p* < 0.05). However, no statistically significant difference was observed among study groups in the following days of the cold storage (Fig. 3).

#### Sperm acrosome integrity and capacitation status

**Fig. 4:**
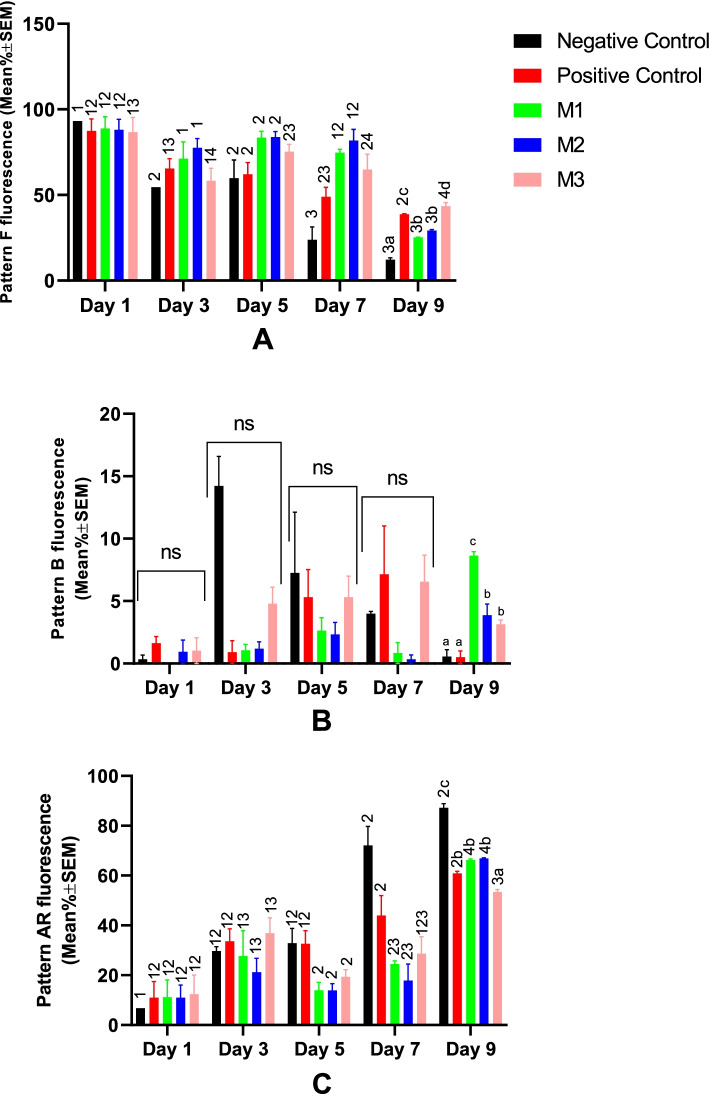
Changes in canine sperm acrosome status were detected by the CTC staining method during cold storage (5 °C) in the extenders containing different levels of melatonin versus the controls. A two-way repeated-measures ANOVA statistical test was used to make a comparison between treatment groups at different times. Pairwise comparisons with LSD confidence interval adjustments were carried out to examine the simple effects for the time and study groups. The different letters and numbers in the bar superscripts indicate a statistically significant difference (*P* ≤ 0.05) between study groups at the specified time point and between time points at the specified group, respectively. Negative Control (without Melatonin or DMSO), Positive Control (containing DMSO), M1 (containing 0.5 mM Melatonin), M2 (containing 1 mM Melatonin), and M3 (containing 2 mM Melatonin), ns, non-significant. All numbers are presented as Means±SEM. Pattern F = non-capacitated with intact acrosome, pattern B = capacitated with intact acrosome, and Pattern AR = reacted/damaged acrosome, CTC=Chlortetracycline

All groups showed a significant decrease in the acrosome integrity at Day 9 of the cold storage (Fig. 4A, C). From the start of the cold storage to Day 7, no statistically significant difference was observed among study groups for spermatozoa acrosomal patterns. However, M1 and M2 groups resulted in significantly higher acrosome integrity at Day 9 (Fig. 4A, B). The percent of sperm cells with CTC pattern B (capacitated) was not statistically different between groups through Day 1 to Day 7 of the cold storage. However, on Day 9, it was higher in all melatonin-treated groups compared with NC and PC (Fig. 4B).

#### Sperm DNA integrity

**Fig. 5:**
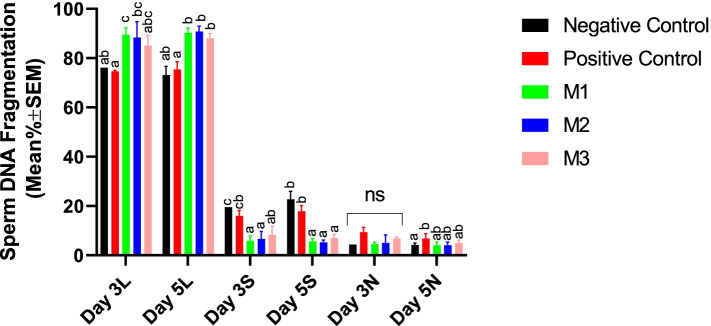
Changes in canine sperm DNA fragmentation were detected by SCDT during cold storage (5 °C) in the extenders containing different levels of melatonin versus the controls. A two-way repeated-measures ANOVA statistical test was used to make a comparison between treatment groups at different times. Pairwise comparisons with LSD confidence interval adjustments were carried out to examine the simple effects for the time and study groups. The different letters in bar superscripts indicate a statistically significant difference (*P* ≤ 0.05) between study groups at Day 3 or 5. Negative Control (without Melatonin or DMSO), Positive Control (containing DMSO), M1 (containing 0.5 mM Melatonin), M2 (containing 1 mM Melatonin), and M3 (containing 2 mM Melatonin), ns, non-significant. All numbers are presented as Means±SEM. L = Large Halo (No DNA fragmentation), S=Small Halo (Partial DNA fragmentation), and N=No Halo (Complete DNA fragmentation), SCDT = Sperm Chromatin Dispersion Test

Results of the current research indicated that supplementation with melatonin significantly improved the sperm DNA integrity as evidenced by increasing the percent of cells with non-fragmented DNA while reducing the percent of cells with partially-fragmented DNA. However, the protective effects were not dose-dependent (Fig. 5).

#### Sperm MDA level at day 5

**Fig. 6:**
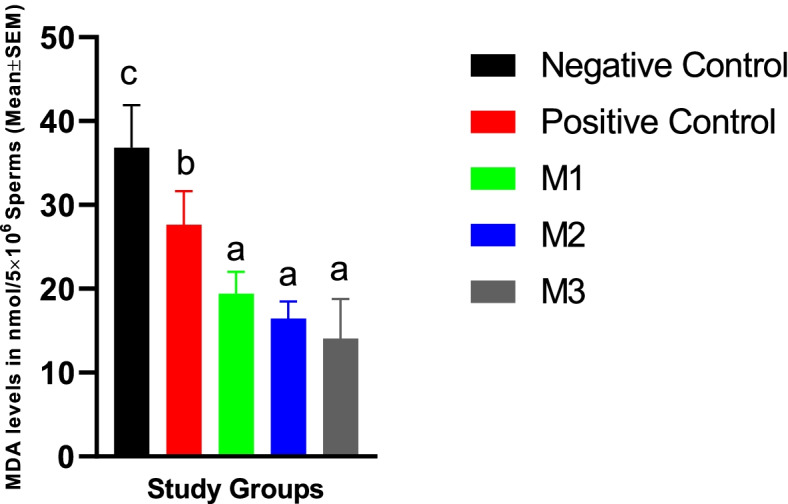
Canine spermatozoa MDA levels at Day 5 of cold storage in different study groups. Similar letters in bar superscripts indicate a statistically non-significant difference (*P* > 0.05). LSD posthoc test was used to perform pairwise comparisons. A one-way ANOVA statistical test was used to make a comparison between treatment groups. Negative Control (without Melatonin or DMSO), Positive Control (containing DMSO), M1 (containing 0.5 mM Melatonin), M2 (containing 1 mM Melatonin), and M3 (containing 2 mM Melatonin). All numbers are presented as Mean% ± SEM

The addition of melatonin at all concentrations used in the current study resulted in a significant decrease in the sperm MDA at day 5 of the cold storage. However, the effects were not dose-dependent (Fig. 6). The DMSO alone was also able to reduce the MDA level, although to a level higher than melatonin groups. The highest level of MDA was observed in the negative control group (Fig. 6).

### Effects of melatonin supplementation on canine sperm freezing

#### Sperm motility parameters

Supplementation of the freezing medium with melatonin at either 1 or 2 mM (M2 and M3 group) did not affect the post-thaw sperm TM and PM values compared with the NC. Melatonin at 0.5 mM concentration (M1) reduced the post-thaw sperm TM and PM values compared with the NC, M2, and M3 groups. The addition of DMSO alone (PC) reduced the post-thaw sperm PM significantly, while, the post-thaw sperm TM was not affected compared with the NC. Melatonin at M2 and M3 groups did not affect the sperm velocity parameters (VCL, VAP, and VSL) compared with the NC and PC groups. However, they were significantly lower in the M1 group. Post-thaw ALH, BCF, and MAD values were also significantly lower in the M1 group compared with the M2 and M3. The STR value was significantly higher in the NC compared with the others. The LIN value in the NC group was significantly higher than other groups except for the M2. The WOB values were not affected by the treatments in the current study (Table 4).

**Table 4:** Motility parameters of the canine post-thaw sperm after cryopreservation in extenders containing different levels of melatonin versus the controls

	Group				
	Negative Control	Positive Control	M1	M2	M3
**TM**	49.52 ± 5.69 ^b^	35.82 ± 7.65 ^ab^	25.57 ± 3.21 ^a^	54.64 ± 8.24 ^b^	50.45 ± 6.90 ^b^
**PM**	21.41 ± 3.06 ^d^	13.07 ± 2.58 ^bc^	6.76 ± .77 ^ab^	20.9 ± 2.35 ^d^	18.90 ± 1.78 ^cd^
**VCL**	20.03 ± 2.68 ^c^	13.70 ± 3.26 ^ac^	8.35 ± 1.00 ^a^	18.27 ± 3.12 ^bc^	19.76 ± 2.25 ^bc^
**VAP**	11.00 ± 1.24 ^c^	7.08 ± 1.61 ^ac^	4.32 ± .55 ^a^	10.50 ± 2.17 ^bc^	10.14 ± .97 ^bc^
**VSL**	7.65 ± .92 ^c^	4.57 ± 1.00 ^ac^	2.65 ± .35 ^a^	6.67 ± 1.31 ^bc^	6.50 ± .45 ^bc^
**ALH**	1.30 ± .19 ^ab^	1.05 ± .22 ^ab^	.73 ± .09 ^a^	1.47 ± .31 ^b^	1.38 ± .20 ^b^
**BCF**	2.23 ± .39 ^ab^	1.47 ± .43 ^ab^	.75 ± .10 ^a^	2.31 ± .60 ^b^	2.39 ± .33 ^b^
**MAD**	23.72 ± 3.59 ^ab^	15.70 ± 4.16 ^ab^	8.99 ± 1.07 ^a^	24.93 ± 6.68 ^b^	23.26 ± 2.65 ^b^
**STR**	69.48 ± 2.23 ^b^	64.68 ± 1.24 ^a^	61.05 ± .70 ^a^	64.04 ± 1.10 ^a^	64.62 ± 1.83 ^a^
**LIN**	38.61 ± 2.11 ^b^	33.63 ± 1.25 ^a^	31.52 ± .60 ^a^	36.25 ± 2.08 ^ab^	33.43 ± 1.61 ^a^
**WOB**	55.47 ± 1.65	51.94 ± .98	51.62 ± .52	56.65 ± 3.41	51.65 ± 1.12

A one-way ANOVA statistical test was used to make a comparison between treatment groups. LSD posthoc test was used to perform pairwise comparisons. Negative Control (without Melatonin or DMSO), Positive Control (containing DMSO), M1 (containing 0.5 mM Melatonin), M2 (containing 1 mM Melatonin), and M3 (containing 2 mM Melatonin). different letters in the superscripts indicate a statistically significant difference (*P* ≤ 0.05) between study groups. All numbers are presented as Means±SEM. *VCL* Velocity Curvilinear (μm/s), *VAP* Velocity Average Path (μm/s), *VSL* Velocity Straight Path (μm/s), *ALH* Amplitude of Lateral Head Displacement (μm), *BCF* Beat Cross Frequency (Hz), *MAD* Mean Angular Displacement (°), *STR* Straightness (%), *LIN* Linearity (%), and *WOB* Wobble (%)

#### Sperm viability and morphology

**Fig. 7:**
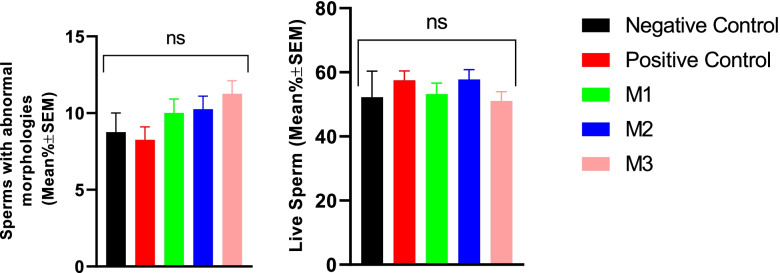
Viability and abnormal morphology of the canine post-thaw sperm after cryopreservation in extenders containing different levels of melatonin versus the controls. A one-way ANOVA statistical test was used to make a comparison between treatment groups and an LSD posthoc test was used to perform pairwise comparisons. Negative Control (without Melatonin or DMSO), Positive Control (containing DMSO), M1 (containing 0.5 mM Melatonin), M2 (containing 1 mM Melatonin), and M3 (containing 2 mM Melatonin). ns, a non-significant difference. All numbers are presented as mean live or morphologically abnormal sperm % ± SEM. *P* values ≤0.05 were considered statistically significant

Results of the current study showed that the addition of the melatonin to freezing extenders did not affect either the spermatozoa post-thaw viability or abnormal morphology rates (Fig. 7).

#### Sperm membrane functional integrity

**Fig. 8:**
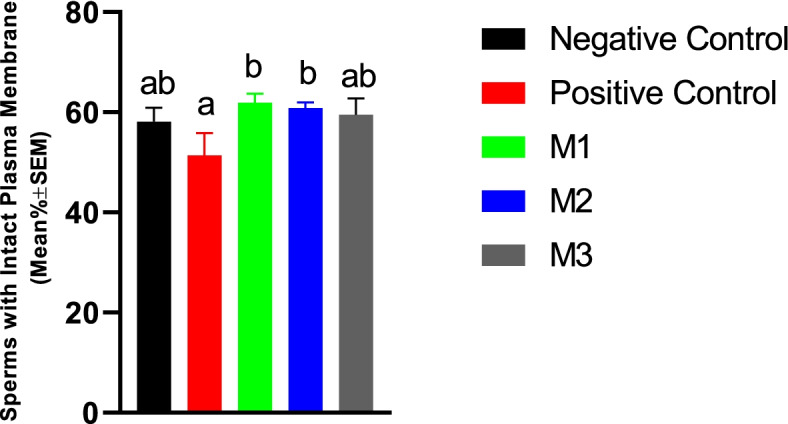
Plasma membrane functional integrity of the canine post-thaw sperm evaluated by HOS test after cryopreservation in extenders containing different levels of melatonin versus the controls. The One-way ANOVA statistical test was used to make a comparison between treatment groups and the LSD posthoc test was used to perform pairwise comparisons. Different letters in bar superscripts indicate a statistically significant difference (*P* ≤ 0.05). Negative Control (without Melatonin or DMSO), Positive Control (containing DMSO), M1 (containing 0.5 mM Melatonin), M2 (containing 1 mM Melatonin), and M3 (containing 2 mM Melatonin). All numbers are presented as Mean% ± SEM, HOS=Hypo-Osmotic Swelling Test

Our results showed that the sperm plasma membrane biochemical integrity was not affected by melatonin supplementation. However, it was lower in the PC group compared with the M1 or M2 groups (Fig. 8).

#### Sperm acrosome integrity and capacitation status

**Fig. 9:**
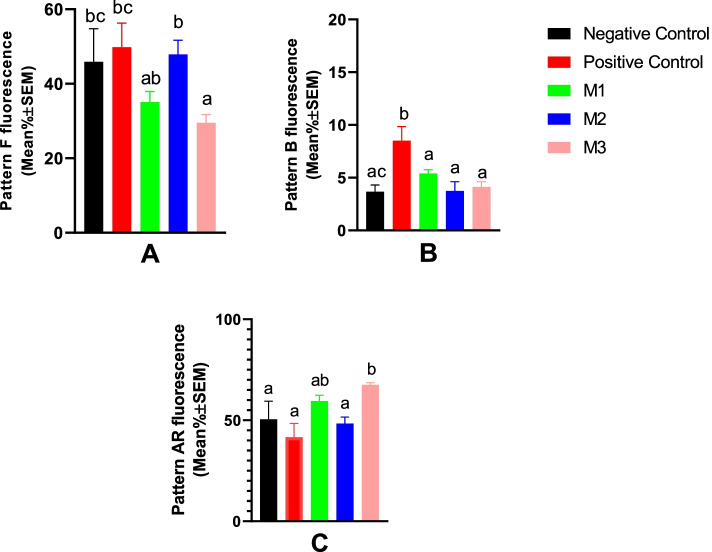
Acrosome integrity and capacitation status of the canine post-thaw sperm evaluated by CTC staining after cryopreservation in extenders containing different levels of melatonin versus the control groups. One-way ANOVA statistical test was used to make a comparison between treatment groups and the LSD posthoc test was used to perform pairwise comparisons. Different letters in bar superscripts indicate a statistically significant difference (*P* ≤ 0.05). Negative Control (without Melatonin or DMSO), Positive Control (containing DMSO), M1 (containing 0.5 mM Melatonin), M2 (containing 1 mM Melatonin), and M3 (containing 2 mM Melatonin). All numbers are presented as Means±SEM. Pattern F = non-capacitated with intact acrosome, pattern B = capacitated with intact acrosome, and Pattern AR = reacted/damaged acrosome, CTC=Chlortetracycline

Compared with the NC and PC groups, melatonin at 2 mM concentration resulted in a significant decrease in the percent of post-thaw spermatozoa with intact acrosomes while significantly increasing the cells with damaged/reacted acrosomes (Fig. 9A and C). Freezing-induced capacitation-like changes (pattern B) were higher in the PC group compared with the others, which could be controlled by melatonin addition irrespective of the concentration used (Fig. 9).

#### Sperm DNA integrity

**Fig. 10:**
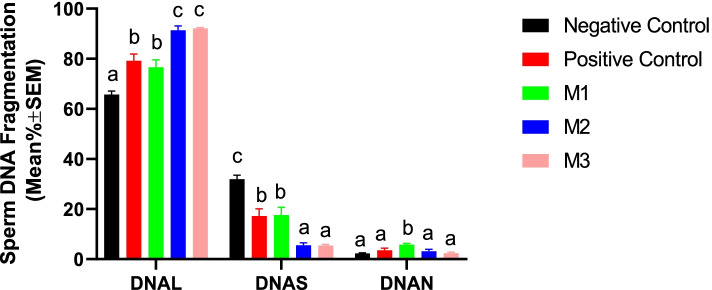
Changes in canine post-thaw sperm DNA Fragmentation were detected by SCDT after cryopreservation in the extenders containing different levels of melatonin versus the controls. A two-way repeated-measures ANOVA statistical test was used to make a comparison between treatment groups within different times and the LSD posthoc test was used to perform pairwise comparisons. Different letters in bar superscripts indicate a statistically significant difference (*P* ≤ 0.05). Negative Control (without Melatonin or DMSO), Positive Control (containing DMSO), M1 (containing 0.5 mM Melatonin), M2 (containing 1 mM Melatonin), and M3 (containing 2 mM Melatonin). All numbers are presented as % ± SEM. L = Large Halo (No DNA fragmentation), S=Small Halo (Partial DNA fragmentation), and N=No Halo (Complete DNA fragmentation), SCDT = Sperm Chromatin Dispersion Test

Our research showed that the addition of the melatonin at 1- and 2-mM concentrations could significantly protect the frozen-thawed spermatozoa against freezing-induced DNA fragmentation. Melatonin in the M2 and M3 groups could also significantly reduce the cells with partially- and totally-fragmented DNA (Fig. 10).

#### Post-thaw sperm MDA level

**Fig. 11:**
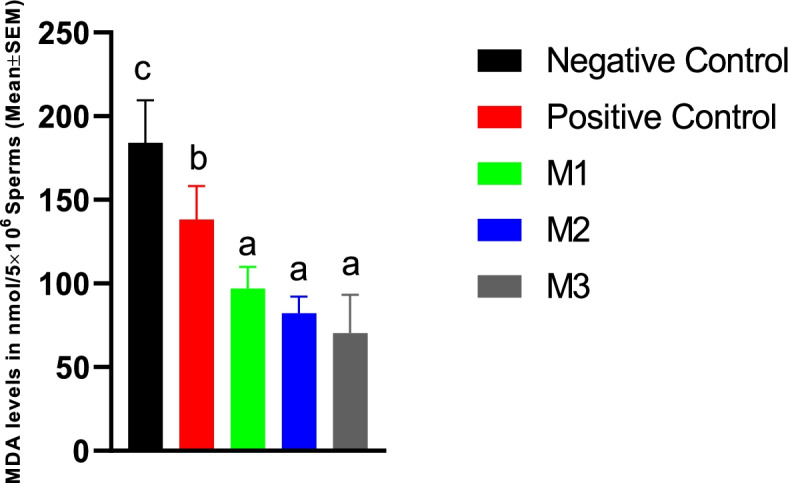
Canine frozen-thawed spermatozoa MDA levels in the extenders containing different levels of melatonin versus the controls. LSD posthoc test was used to perform pairwise comparisons. A one-way ANOVA statistical test was used to make a comparison between treatment groups. Different letters in bar superscripts indicate a statistically significant difference (*P* ≤ 0.05). Negative Control (without Melatonin or DMSO), Positive Control (containing DMSO), M1 (containing 0.5 mM Melatonin), M2 (containing 1 mM Melatonin), and M3 (containing 2 mM Melatonin). All numbers are presented as Mean% ± SEM

Our results indicated that the freezing process could increase the sperm MDA level significantly higher than day 5 cold-stored spermatozoa levels. The addition of melatonin at all concentrations used in the current study resulted in a significant decrease in the post-thaw sperm MDA content. However, the effects were not dose-dependent. The DMSO alone was also able to reduce the MDA level, although to a level higher than melatonin groups. The highest level of MDA was observed in the negative control group (Fig. 11).

#### MTT reduction assay

**Fig. 12:**
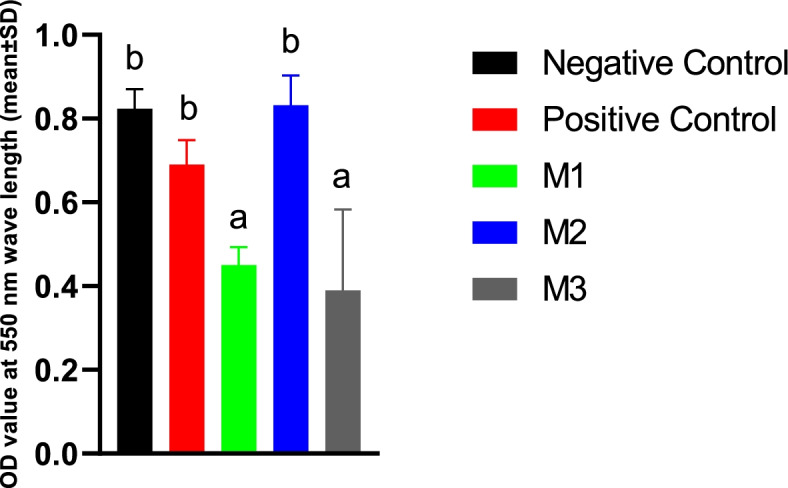
MTT reduction assay results measured by ELISA UV-VIS microplate reader at a wavelength of 550 nm in the extenders containing different levels of melatonin versus the controls. Absorbance values are differences of ODs at the start and after an incubation period of 1 h at 37 °C. One-way ANOVA and LSD posthoc statistical tests were used to make a comparison between treatment groups. Different letters in bar superscripts indicate a statistically significant difference (*P* ≤ 0.05). Negative Control (without Melatonin or DMSO), Positive Control (containing DMSO), M1 (containing 0.5 mM Melatonin), M2 (containing 1 mM Melatonin), and M3 (containing 2 mM Melatonin). All numbers are presented as mean% ± SD, *n* = 4

Melatonin at either 0.5 mM or 2 mM concentrations showed detrimental effects on the sperm metabolic and mitochondrial activity as evidenced by a significant decrease in the MTT reduction rate. However, the spermatozoa with 1 mM concentration had the same metabolic activity as the PC or NC groups (Fig. 12).

## Discussion

In the present study, we evaluated the in vitro effects of melatonin on canine spermatozoa preservation both during cold storage and freeze-thawing. In the cooling part, our results indicated that melatonin at 0.5, 1- or 2-mM concentrations could preserve significantly higher sperm total motility after 4 days of cold storage (5 °C). Similarly, melatonin had significant protective effects on the cooled sperm DNA integrity either on Day 5 or Day 9 of the study. Several previous studies have reported an increase in the spermatozoa reactive oxidative species content concurrent with a decrease in antioxidant capacity (oxidative stress) during long-term cold storage in different animal species [12]. Therefore, supplementation of antioxidants could ameliorate sperm oxidative stress and improve sperm quality measures during cold storage [12]. In confirmation, we showed that melatonin supplementation could protect canine sperm acrosome and DNA integrity which are important determinants for the sperm’s future fertility [13]. As we observed a simultaneous decline in the Day 5 MDA levels in the treatment groups, the protective effects of melatonin might have come from its antioxidant properties. In our study, the 1- and 2-mM concentrations (M2 and M3) could result in significantly better TM, PM, sperm viability, and acrosome integrity values after 9 days of cold storage. Interestingly, the DMSO alone (PC group) has also been able to improve sperm viability, acrosome integrity with a simultaneous decline in the MDA level which probably comes from its antioxidant effects. Therefore, at least some part of protective effects in the M2 and M3 groups after long-term cold storage has been caused by the DMSO itself.

Among domestic species, cold storage of the boar, stallion, and canine spermatozoa is a common semen preservation method. In a previous study [14] melatonin at 1 μM (34 mM stock solution in ethanol) was added to a commercial extender for boar spermatozoa and was kept at 17 °C for up to 7 d. Although they showed that melatonin increased the proportion of live sperm with an intact acrosome, it failed to enhance the quality of refrigerated boar semen and even in some measures caused negative effects. Another study was to evaluate the effects of melatonin on the quality of Caspian stallion spermatozoa during cooling preservation [15]. The results showed that semen extender containing 1.5 mM melatonin improved sperm quality of Caspian stallion during 48 h storage in cool conditions. Another research group added melatonin at 1 mM and 2 mM to the cooling extender for ram spermatozoa [16]. They reported that the sperm quality was better in those treatments supplemented with melatonin after 48 h of cold storage.

In the freezing section of the current research, melatonin at either 1- or 2-mM concentrations could not improve the sperm post-thaw TM and PM, while they improved sperm DNA integrity. Also, the plasma membrane functional integrity and sperm velocity parameters were not affected. Similar to our results, several researchers have reported the protective effects of melatonin added to the sperm freezing media in humans [8] and different animal species [9]. Literature review shows wide variability in the range and the best protective concentrations of melatonin on the frozen-thawed spermatozoa in several animal species. The results are different even between individuals of the same species [9]. Also, studies are different in the type of solvent medium they have used to deliver melatonin to the sperm media. Although the lipophilic property of melatonin makes it easier to pass through cellular barriers, it carries the disadvantage of requiring an appropriate amphoteric solvent before it can be used for in vitro studies. DMSO and ethanol are the two most common solvents for melatonin supplementation studies. The DMSO as the melatonin carrier exerted positive effects in the cooling part of our study. In contrast, in the freezing part of our research, the DMSO alone declined the post-thaw sperm PM significantly, although it reduced the level of sperm membrane lipid peroxidation and even improved the post-thaw sperm DNA integrity compared to the negative control. However, the DMSO negative effects were reversed by concurrent melatonin supplementation at 1- and 2-mM concentrations. Some previous researches have also shown that both DMSO and ethanol as carriers could have some negative unwanted effects on the sperm cells even at very low quantities [9].

The effects of melatonin on canine ejaculated frozen-thawed spermatozoa were not subjected to extensive research before. Furthermore, its potential protective effects on canine sperm cells during long-term cold storage had not been studied yet. Varesi et al., 2014, evaluated the potential protective effects of melatonin on the post-thaw epididymal sperm quality in dogs. They showed that supplementation of melatonin to the sperm freezing extender (1 mM) did not affect sperm motility, acrosome, and DNA integrity. Unfortunately, the type of melatonin solvent was not reported in the study. In another study on Bulldogs [10], the authors tested the effect of melatonin in the freezing medium on sperm cryo-survival. Similar to us, they used DMSO as the melatonin solvent. However, in contrast to us, they prepared the melatonin (0.0, 0.0005, 0.002, and 0.0035 mol/L) and control (DMSO + PBS (1 + 9, v/v)) groups so that all of them received the same concentrations of DMSO. They reported that melatonin supplementation could not have a significant effect on post total motility and plasma membrane integrity. However, it could reduce the percentage of acrosome-damaged sperms following cryopreservation, with the 0.002 and 0.0035 mol/L concentrations having better results.

In a previous study [17], melatonin at 0.1 mM did not affect the bovine sperm post-thaw kinematic parameters. However, it could provide significant protection on the sperm ultrastructure. They showed that higher concentrations (0.2 and 0.25 mM) were associated with the highest plasma and acrosome membrane damage. In the current study, we observed a negative effect on sperm post-thaw acrosome integrity by melatonin supplementation at 2 mM concentration. Another study [18] concluded that the most protective concentration of melatonin in the freezing extender based on their post-thaw evaluations of the bull sperm was 2 mM. While the 4 mM concentration caused negative effects. Similarly, the addition of 1.25 mM of the melatonin in Crioulo stallion sperm cells showed to have a protective effect on the sperm cell during cryopreservation. However, the sperm post-thaw motility was not affected and higher concentrations exerted negative effects [19].

Although we showed that the addition of melatonin to the in vitro canine sperm media improves sperm quality markers, the positive effects on the final sperm fertility need to be confirmed through in vitro or in vivo fertility trials. For example, it is shown that free radicals play role in sperm capitation in multiple species [20]. Therefore, excessive neutralization of the free radicals via the addition of antioxidants could probably interfere with the sperm capacitation process and hampers oocyte fertilization. It is recommended that the effects of melatonin on the canine sperm capacitation process be checked under in vitro conditions followed by in vitro fertilization and early embryonic development.

In conclusion, supplementation of canine sperm cooling and freezing media with melatonin at 1- or 2-mM concentrations could improve cold stored and frozen-thawed sperm quality markers which may lead to an improvement in the spermatozoa future fertility after insemination.

## Methods

### Animals and ethics

Seven adult large mixed-breed dogs (with mean’s ± SD for the body weight and age as 19.85 ± 3.7 kg and 2.57 ± 0.53 years, respectively) were used for semen collections in the current research. The sheltered animals were kept under controlled environmental conditions (25 °C, 12:12 h. of light: dark cycles) and fed a balanced commercial canine feed (Nutripet™, Behintash Co., Karaj, Iran) and had free access to water. The present study was carried out from October to December 2020. All designs and procedures in the current research complied with the ARRIVE guidelines and followed the UK Animals (Scientific Procedures) Act, 1986 and associated guidelines, EU Directive 2010/63/EU for animal experiments. Our study was also approved by the Iranian laboratory animal ethics framework under the supervision of the Iranian Society for the Prevention of Cruelty to Animals and Shiraz University Research Council (IACUC no: 4687/63).

### Semen collection and preparation

Semen collections were regularly carried once every 3 days. Semen samples (pre-sperm rich and sperm-rich fractions) were collected via manual stimulation using a latex collecting cone attached to 15 ml sterile pre-warmed plastic tubes (Falcon, USA). All samples were collected in 45 min and transferred to the laboratory while kept at 37 °C in a portable electronic warmer within 5 min. Upon arrival, the semen samples were evaluated subjectively for sperm mass motility. Only samples with more than 60% mass sperm motility were used in the downstream procedures. Next, the semen samples were centrifuged (600×g, 10 min, at room temperature) and 90% of supernatant seminal plasma was removed. The remaining sperm pellets were pooled together and used for cooling or freezing parts of the current study. The current study was replicated five times in the cooling and five times in the freezing sections, independently.

### Part a. cooling study

#### Preparation of cooling semen extender and melatonin supplementation

We used a non-commercial Tris-Citric Acid-Fructose extender base (Tris 3.025 g, Citric acid 1.7 g, Fructose 1.25 g, Crystalline Penicillin G 100000 IU, Streptomycin sulfate 150 mgr., milli-Q highly purified water q.s. to 100 ml final volume, pH = 6.77, Osmolality = 360 mosmol/kg water at 25 °C) supplemented with fresh albumin-free chicken egg yolk (15%, v/v) to store semen under cold (5 °C) condition. The melatonin stock solution was prepared by dissolving 34.8 mgr. Melatonin powder (Sigma Aldrich, M5250) in 20 μl DMSO (Dimethyl Sulfoxide, Sigma Aldrich, D8418). Melatonin working solution was prepared by diluting 10 μl of melatonin stock solution to 500 μl in sterile double distilled highly purified water. Each independent replicate included five groups: Negative Control (NC, no supplementation), Positive Control (PC, DMSO only), 0.5 mM melatonin (M1), 1 mM melatonin (M2), and 2 mM melatonin (M3). Volumes of 5, 10, and 15 μl of melatonin working solution were added to 3 ml extended sperm suspensions (50 million/ml) in M1, M2, and M3 melatonin supplementation groups, respectively. The DMSO alone was diluted similar to melatonin groups and a volume of 15 μl was added to the PC group. Then, samples were slowly cooled down to 5 °C in glass beakers (250 ml) containing 150 ml room temperature tap water for 3 h and stored at the same temperature for 9 days. Sperm samples for the laboratory procedures were collected on Day 1 (as soon as the sperm suspension first reached 5 °C), Day 3, Day 5, Day 7, and Day 9. All cooled semen samples were warmed up in a 37 °C water bath for 5 min before being used for the downstream sperm quality measurements.

### Part B. freezing study

#### Preparation of freezing semen extender and melatonin supplementation

We employed a two-step freezing method in a Tris-Citrate-Fructose extender supplemented with fresh albumin-free chicken egg yolk (25%, v/v) to freeze the recovered sperm cells. The sperm density was set to 400 million/ml using room temperature (25 °C) part A freezing medium (Tris 3.025 g, Citric acid 1.7 g, Fructose 1.25 g, Crystalline Penicillin G 100000 IU, Streptomycin sulfate 150 mgr., milli-Q highly purified water q.s. to 80 ml final volume, pH = 6.77, Osmolality = 460 mosmol/kg water) and cooled to 5 °C in a glass beacon containing 150 ml tap water (25 °C) in the refrigerator over 3 h. Next, an equal volume of the pre-cooled (5 °C) part B freezing medium (the same ingredients as part A, supplemented with glycerol (10%, v/v) and 0.5% SDS (w/v) was added to the semen suspension and left at the same temperature for 45 min for glycerol equilibrium. Then, the extended semen was loaded into sterile plastic half-ml semen straws (IMV, France) and sealed at the open end with polyvinyl Alcohol powder. Loaded straws were left over liquid nitrogen (LN) four centimeters above the surface for 20 min. Finally, semen straws were plunged into the LN and transferred to the LN storage tank. The straws were stored at the LN tank for at least 3 weeks before thawing in a 37 °C water bath for 30 s. Within each repeat, at least three straws from each study group were thawed and pooled together for downstream procedures to control for the freezing procedure variations. Each freezing replicate included five groups: Negative Control (NC, no supplementation), Positive Control (PC, DMSO only), 0.5 mM melatonin (M1), 1 mM melatonin (M2), and 2 mM melatonin (M3). Melatonin preparation and supplementations to both parts A and B freezing extenders were carried out as explained under Part A. section in the current paper.

### Sperm quality measures

#### Computer-assisted sperm motion analysis (CASA)

The percent of total motile spermatozoa (TM), motile sperms with rapid progressive movement (PM), and sperm velocity parameters including curvilinear path velocity (VCL, mm/s), straight-line path velocity (VSL, mm/s), and average path velocity (VAP, mm/s) were evaluated objectively by a CASA system (Houshmand Fanavar, Tehran, Iran) equipped with a bright-field light microscope and a preheated (37 °C) analysis chamber (Sperm360 Labs). Sperm velocity ratios, Straightness and Linearity, were also calculated. A minimum of 400 cells in 4 random microscopic fields (× 100 magnification) was counted. A sperm cell was classified as immotile when the VAP value was less than 10 mm/second, and the CASA frame rate was set to 50 Hz. The sperm density of frozen-thawed samples was set to 100 million/ml with the pre-warmed freezing extender base (under Part B. section in the current paper) before CASA analysis.

#### Sperm viability and morphology

The sperm viability and morphology were determined through supravital Eosin-Nigrosine staining. A five-microliter semen sample was mixed with an equal volume of the stains on a warm microscope slide. Next, a smear was prepared and dried immediately. Viability and morphology were evaluated for at least 200 cells at × 1000 magnification under a bright-field light microscope (Olympus, Japan). Exclusion of eosin stain due to membrane integrity was considered as viable status. Morphological abnormalities were categorized as normal and abnormal groups [21].

##### Sperm membrane biochemical integrity

The biochemical integrity of the sperm plasma membrane was assessed by a hypo-osmotic sucrose solution (150 mOsm/L). Ten-μl samples of the semen were added to 90 μl prewarmed hypo-osmotic solution and left on the heat block at 37 °C for 30 min. Next, a wet mount was prepared and evaluated at × 400 magnification under a bright-field light microscope (Olympus, Japan). Sperm cells with swelling/coiling of the tail were considered to have intact plasma membrane [22].

#### Sperm acrosome integrity and capacitation status

Calcium-dependent capacitation-related changes and sperm acrosome status were determined via chlortetracycline (CTC) fluorescence assay. Five microliters of CTC working solution (750 mmol/l CTC in 130 mmol/l NaCl, 5 mmol/l cysteine, 20 mmol/l Tris-HCl, pH = 7.8) was mixed with an equivalent volume of the semen sample and left for 5 s. Next, cells were fixed in 5 μl of 12.5% Glutaraldehyde (Sigma) prepared in 0.5 mol/l Tris-HCl (pH = 7.4). The sperm suspension was covered by a coverslip and sealed with nail polish to avoid evaporation. The slides were kept in a dark and cold chamber and evaluated under a fluorescent light microscope (ZEISS Primo Star iLED, Carl Zeiss MicroImaging GmbH, Germany) within 30 min with × 1000 magnification [22]. Three distinct fluorescence patterns on the sperm head were observed and classified as: (F), Uniform bright fluorescence over the whole head (un-capacitated sperms with intact acrosome), (B), Fluorescence-free band in the post-acrosome region (capacitated sperms), (AR), No/Dull fluorescence over the whole head except for a thin punctate band of fluorescence along the equatorial segment (acrosome-reacted or acrosome-damaged sperms) [22].

### Sperm DNA integrity

Sperm DNA fragmentation (double-strand breaks) was assessed by sperm chromatin dispersion test using a commercial kit (Houshmand Fanavar Co, Tehran, Iran). Briefly, the sperm sample (25 μl) was mixed with 1% low-melting-point aqueous agarose at 37 °C and were transferred onto a glass slide pre-coated with dried 0.65% standard agarose, covered with a coverslip and left to solidify at refrigerator temperature. Next, coverslips were removed, and slides were soaked in the acid denaturation solution (0.08 N HCl) for 7 min at room temperature in the dark. Then, the slides were coated with neutralizing and lysing solution (0.4 M Tris, 0.8 M DTT, 1% SDS, and 50 mM EDTA, pH 7.5) for 14 min at room temperature. Slides were thoroughly washed in tap water for 5 min, dehydrated in sequential 70, 90, and 100% ethanol baths (2 min each), and air-dried. Cells were stained with the Diff-Quick reagents for brightfield microscopy. A total of 200 sperms were included in the calculation of spermatozoa with intact (the large halo around the sperm head), partially (the small halo around the sperm head), and fragmented DNA (no halo around the sperm head) in mean percentages [23].

### MDA level

We used a thiobarbituric acid (TBA) colorimetric quantitative assay using a commercial kit (ZellBio, Germany) which detects MDA-TBA adduct formed by the reaction of the MDA and thiobarbituric acid (TBA) under high temperature. Malondialdehyde is measured in acidic media and heat (90–100 °C) colorimetrically at 535 (530–540 nm). Briefly, appropriate aliquots of semen samples (containing 5 × 10^6^ sperm cells) were collected in sterile cryotubes, snap-frozen in liquid nitrogen, and kept at − 20 °C until further analysis. Next, the samples were warmed/thawed and sperm cells were disrupted by repeated freeze-thawing in liquid nitrogen. After the addition of TBA to the supernatant and 60 min incubation period in boiling water, the supernatant was collected after centrifugation (3000 rpm, 10 min) and the level of absorbance (OD) at 550 nm wavelength was read using an enzyme-linked immunosorbent assay plate reader. The absolute levels of MDA were calculated by comparing the standard and sample OD values. The commercial kit intra- and inter-assay CV (%) was reported to be 5.8 and 7.6, respectively.

### MTT reduction assay

Post-thaw sperm metabolic and mitochondrial activity was assessed through a comparative MTT reduction assay. The sperm oxidoreductase enzymes reduce the tetrazolium dye MTT 3-(4,5-dimethylthiazol-2-yl)-2,5-diphenyltetrazolium bromide to its insoluble formazan, which has a purple color and can be quantified through spectrophotometry. The post-thaw pooled sperm suspension from each group was immediately diluted with the pre-warmed (37 °C) part A extender base (all ingredients except for the egg yolk) to reach the sperm density of 50 million/ml. Next, 100 μl diluted samples were transferred to sterile ELISA microplate wells and mixed with pre-warmed 10 μl MTT working solution (5 mgr. MTT/ml of PBS) in each well. The loaded microplate was read by an ELISA UV-VIS microplate reader at a wavelength of 550 nm and then incubated at 37 °C in a shaker incubator for 30 min (for the cold stored spermatozoa) or 1 h (for the frozen-thawed spermatozoa). Finally, the microplate was read again by the microplate reader at the same wavelength. A part of diluted post-thaw semen was centrifuged at 2000 g for 5 min and the supernatant (100 μl, supplemented with 10 μl MTT solution) was used as a blank sample for the spectrophotometry. The OD (optic density) difference between the start and the end of the incubation period was calculated and reported in figures after statistical analyses [22, 24].

### Statistical analyses

After evaluation of the test assumptions (normal distribution of the dependent variable for each category of the independent variable using Shapiro-Wilk test and homogeneity of variances using Levene’s test), ANOVA statistical test was used to make a comparison between treatment groups. LSD posthoc test was used to perform pairwise comparisons. *P* ≤ 0.05 was considered a statistically significant difference. All Statistical procedures were carried out using SPSS software, IBM Corp. Released 2016. IBM SPSS Statistics for Windows, Version 24.0. Armonk, NY: IBM Corp. All Figures were created by GraphPad Prism version 8.0.0 for Windows, GraphPad Software, San Diego, California USA.

## Data Availability

The datasets generated and/or analyzed during the current study are available from the corresponding author on request.

## Abbreviations

RM ANOVA: Repeated measure analysis of variance
ALH: Amplitude of Lateral Head Displacement
BCF: Beat Cross Frequency
MAD: Mean Angular Displacement
VCL: Velocity Curvilinear
VAP: Velocity Average Path
VSL: Velocity Straight Path
TM: Total motility
PM: Progressive motility
STR: Straightness
LIN: Linearity
WOB: Wobble
AR: Reacted/damaged acrosome
CTC: Chlortetracycline
SCDT: Sperm Chromatin Dispersion Test
